# Analysis of Sustained Release Behavior of Drug-Containing Tablet Prepared by CO_2_-Assisted Polymer Compression

**DOI:** 10.3390/polym10121405

**Published:** 2018-12-18

**Authors:** Yoshito Wakui, Takafumi Aizawa

**Affiliations:** Research Institute for Chemical Process Technology, National Institute of Advanced Industrial Science and Technology, 4-2-1 Nigatake, Miyagino-ku, Sendai 983-8551, Japan; y-wakui@aist.go.jp

**Keywords:** drug delivery, porous polymer, CO_2_-assisted polymer compression, sustained release, diffusion, porosity, tortuosity, methylene blue

## Abstract

A controlled-release system for drug delivery allows the continuous supply of a drug to the target region at a predetermined rate for a specified period of time. Herein, the sustained release behavior of a drug-containing tablet fabricated through CO_2_-assisted polymer compression (CAPC) was investigated. CAPC involves placing the drug in the center of a nonwoven fabric, sandwiching this fabric between an integer number of nonwoven fabrics, and applying pressure bonding. An elution test, in which the drug-carrying tablet was immersed in water, showed that sustained-release performance can be controlled by the number of nonwoven fabrics covering the top and bottom of the drug-loaded fabric and compression conditions. A model of sustained drug release was formulated to estimate the effective diffusion coefficient in the porous material. Comparative analysis of the bulk diffusion coefficient revealed that the change in diffusion volume due to change in porosity predominates. The tortuosity of the diffusion path was 3–4, and tended to remain almost constant or increase only slightly when the compression rate was increased. These findings show that sustained drug release can be controlled by incorporating the drug into a nonwoven fabric and using the same raw material to encapsulate it.

## 1. Introduction

Plastics are used in various applications because of their lightness and strength [1]. The form of the plastic, such as board, bead, and fiber, varies depending on its usage. In addition, porous plastics are used as cushioning, heat insulators, sound-absorbing materials, and filters, among others. Further, a porous material with a through-hole is also used as a support for holding a drug [2]. Methods of manufacturing such porous polymer materials include foam injection molding [3] and molding with supercritical CO_2_ followed by foaming [4,5].

There are several plastic molding methods such as injection molding, melt spinning, electrospinning, solidification by mixing main and curing agents, and photo formation. In recent years, 3D printers have also been put to practical use in the creation of plastic structures. To bond plastics, the use of adhesives and hot pressing have been proposed. In recent years, Aizawa has developed a CO_2_-assisted polymer compression (CAPC) method, in which plastic fibers are attached by pressing in the presence of CO_2_ at room temperature [6]. The protocol of this method is that the polymer is impregnated with CO_2_, plasticized, and compressed. CO_2_ is known to be easily soluble in polymers [7,8,9] and cause a decrease in the glass transition point and melting point [10,11,12]. Because CO_2_ impregnation does not lower the glass transition point to room temperature, it is possible to plasticize certain polymers to such a degree that they can be compressed. CAPC is a very convenient method of fabricating porous polymer materials because it can be conducted at room temperature, uses only CO_2_, and achieves adhesion without using an adhesive. CO_2_ is released from the fabricated material into the atmosphere to yield an uncontaminated porous body. Moreover, it is possible to control the porosity and pore size distribution by controlling the compression process [13]. The absence of heat and contamination in the process is expected to be most suitable for medicine, especially because the drug-containing sample will not undergo heat denaturation.

An application of plastics in the medical field is drug loading, which uses a water-absorbent polymer and various kinds of tapes such as poultice and bandage [14]. Recently, drug delivery has been extremely important to increasing the added value of a medicine [15]; it is a technique for delivering a designated medicine to a specified location, as well as for continuously supplying a specified medicine in a specified amount. Regarding the loading of the drug on a porous material, control of sustained release is expected because of the nature of the material [16,17,18].

CAPC enables a drug to be easily placed inside a porous material, and when the sample is used as medicine, control of sustained release is expected. This possibility is suggested by the controllability of porosity and pore size, although there have been no reports actually verifying this. In this study, we performed a quantitative analysis of the properties of sustained release of a drug, specifically methylene blue, from samples prepared using nonwoven fabrics. 

Methylene blue has extremely low toxicity and is used as a fungicide in aquariums where tropical fish are bred [19,20,21]. If the tablet can gradually supply methylene blue to an aquarium, it would be possible to leave the tablet in a filter when the fish becomes sick. Therefore, using methylene blue for evaluation is meaningful from the point of view of further application.

First, we investigated the effect of thickness on the elution rate by using samples with different thickness but with the same porosity. Subsequently, the influence of porosity on the elution rate was investigated by fixing the thickness and varying the porosity. Finally, the effect of temperature on the elution rate was investigated by performing the experiment at temperatures between 10 and 60 °C. For these set of experiments, the elution rate was analyzed using a diffusion model, and the influences of the porosity and tortuosity on the diffusion rate in the porous material were discussed.

## 2. Materials and Methods 

### 2.1. Preparation of Drug-Containing Samples

Methylene blue (CAS No. 7220-79-3) was purchased from FUJIFILM Wako Pure Chemical Co. (Osaka, Japan) and used without further purification. The nonwoven fabric (basis weight: about 30 g m^−2^, average diameter: 8 μm) was manufactured by Nippon Nozzle Co., Ltd. (Kobe, Japan) through a melt-blown method using polyethylene terephthalate (PET) pellets (density: 1.34 g mL^−1^, Bell Polyester Products Inc., Product name: TK3, Yamaguchi, Japan). Samples with diameters of 18 or 8 mm were punched out from the fabric.

**Figure 1:**
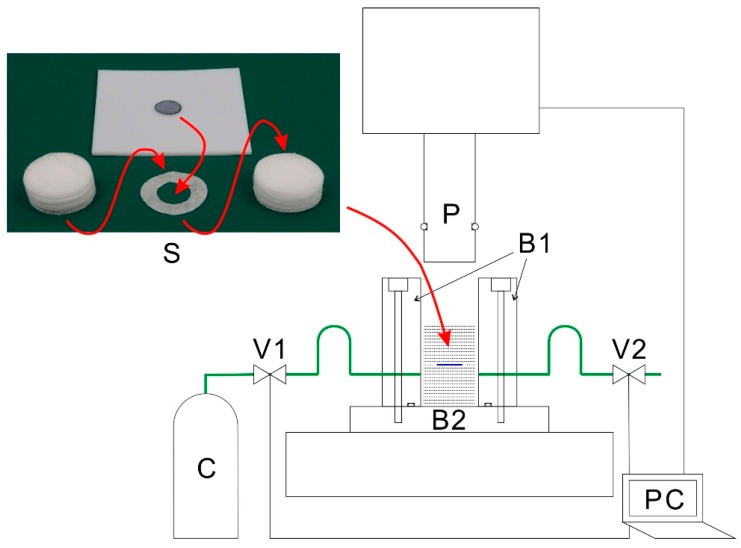
Schematic illustration of the cross-section of the high-pressure vessel used for CO_2_-assisted polymer compression. B1: Body of the high-pressure vessel, B2: Base of the high-pressure vessel, C: CO_2_ cylinder, P: Piston, PC: Laptop computer, S: Sample, V1: Intake valve, and V2: Exhaust valve.

Figure 1 shows the diagram of the cross-section of the high-pressure vessel used to prepare samples via CAPC and the tube connections. The drug-containing sample was prepared by placing a 8-mm round nonwoven fabric on a polytetrafluoroethylene (PTFE) sheet and dropping 10 μL of a 2 wt % solution of methylene blue (in 1:3 ethanol/water). The fabric was then dried and enclosed inside a tablet. The upper left corner of Figure 1 shows the nonwoven fabrics used in the CAPC process. The methylene-blue-loaded fabric (upper middle) was placed inside the 9-mm hole of the donut-shaped nonwoven fabric (lower middle). The top and bottom of this fabric were sealed with the nonwoven fabrics shown on the lower left and right corners of the image. After combining all sheets, the sample underwent CAPC treatment.

In the CAPC process, the piston (P) was first lowered to a certain distance from the bottom of the pressure vessel (B2). CO_2_ was introduced by vapor pressure at a position 1.5 times higher than the press position. The piston and pressure vessel were sealed with the O ring provided on the side of the piston. Because the CO_2_ cylinder (C) connected to the pressure vessel was below the position of the O ring, the CO_2_ introduced into the pressure vessel struck the piston and was supplied to the sample through the gap between the piston and pressure vessel. Subsequently, the introduction and discharge of CO_2_ were repeated three times before replacing air with CO_2_. The piston was lowered to the press position and the sample were pressed for 10 s. After the vessel was evacuated by opening the exhaust valve (V2), the piston was raised and the sample taken out. Because the CAPC process compressed the sheets at the press position, the thickness of the fabricated porous material was almost equal to this position.

Experiments were carried out to evaluate the effect of the diffusion distance using samples with the same porosity but different thicknesses. The effect of porosity dependence of elution was investigated by varying the number of the laminated sheets in the sample.

### 2.2. Porosity Evaluation

Porosity was evaluated in terms of polymer density, weight of the fabric sheet, and sample thickness. The datasheet for the PET pellet indicated that the density of the used polymer is 1.34 g mL^−1^. For a solid without a void, the thickness can be easily calculated using the weight, density, and diameter of the fabric sheet. The thickness at the center of the drug-containing sample was measured using a micrometer screw gauge. Assuming that the center of the drug-loaded nonwoven fabric was also compressed like the sides of the fabric, the thickness of one sheet can be estimated by dividing the measured thickness by the number of nonwoven fabrics. The difference between the thickness of the solid (*L*_solid_) and actual thickness (*L*_sample_) was considered as the total area of the pore, and the porosity α was calculated as α = (*L*_sample_ − *L*_solid_)/*L*_sample_.

### 2.3. Elution Rate Measurement

**Figure 2:**
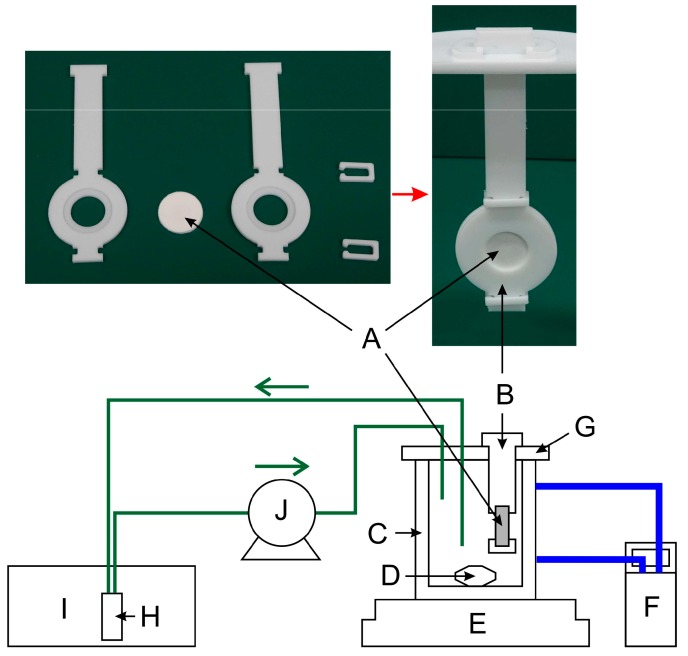
Schematic illustration of the set-up for drug release evaluation. A: Sample, B: Sample holder, C: Jacketed beaker, D: Stirrer bar, E: Magnetic stirrer, F: Circulating water bath, G: Beaker cap, H: Quartz flow cell, I: UV/vis spectrophotometer, and J: Peristaltic pump.

In the elution test, the sample was immersed in water, and the amount of methylene blue eluted in water was measured as concentration change by UV/visible spectroscopy (Shimadzu Co., Kyoto, Japan). The sample holder and experimental set-up are described in Figure 2. The sample holder was made of PTFE to avoid adsorption, and was originally designed by computer numerical control machining. The sample was held in a hole in the sample holder and its outer periphery was sandwiched and fixed with a PTFE clip. 

2-Morpholinoethanesulfonic acid (MES) monohydrate (CAS No. 145224-94-8) was purchased from Dojindo Laboratories (Kamimashikigun, Japan) and used without further purification. The MES buffer solution (1 mM, pH 6.2) was prepared using water purified by ion exchange and distillation. Two hundred milliliters of this solution was placed in a thermostatic jacketed beaker (C). The temperature was adjusted to the specified temperature by a circulating thermostatic water bath (F). The beaker containing the sample holder was confirmed to reach the desired temperature in about 10 min; thus, after more than 1 h, the sample was set in the sample holder and measurement was initiated (defined as time 0). The liquid inside the beaker was stirred at 200 rpm using a magnetic stirrer (E) and circulated to a flow cell (optical path length: 1 cm) at a flow rate of 8.8 mL h^−1^ using a peristaltic pump (J). A low setting was used for the magnetic stirrer to prevent flow inside the drug-containing sample. The flow cell (H) was a commercially available branch-type flow cell for the UV/vis spectrophotometer. The absorbance (λ = 664 nm) of the methylene blue eluted in the aqueous phase was measured at 5-min intervals using a UV/vis spectrophotometer (Shimadzu UV-3150, Kyoto, Japan). 

### 2.4. Estimation of Solubility

An excess amount of methylene blue was added to an aqueous solution containing 1 mM MES buffer and stirred in a water bath at 25 °C. An aliquot of 0.5-mL was taken and centrifuged using a centrifugal filter unit (Durapore PVDF, 0.1 μm, Merck Millipore, Darmstadt, Germany) at 2000 rpm to achieve solid-liquid separation. The liquid phase was diluted 10,000 times; then, the absorbance at 664 nm was measured using a UV/vis spectrophotometer. As a result, the absorbance was almost stabilized after about 2 h. Therefore, after stirring the liquid for more than 12 h under different temperature conditions, visible absorption was similarly measured to evaluate saturation solubility.

## 3. Results and Discussion

**Figure 3:**
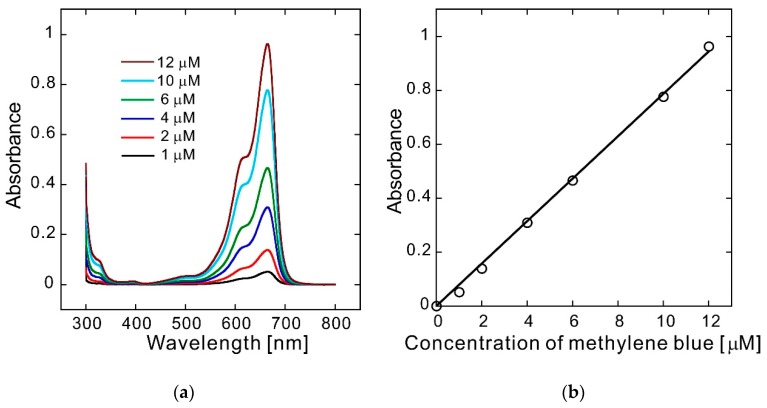
(**a**) UV/vis absorbance spectra of methylene blue and (**b**) absorbance at 664 nm as a function of concentration.

First, validation of the concentration measurement and calibration of the absorbance against concentration were carried out. The observed spectra of methylene blue in the MES buffer solution at steady-state condition are shown in Figure 3a. The linear relationship between the absorbance at 664 nm and methylene blue concentration was maintained up to a concentration of 12 μM (Figure 3b).

**Figure 4:**
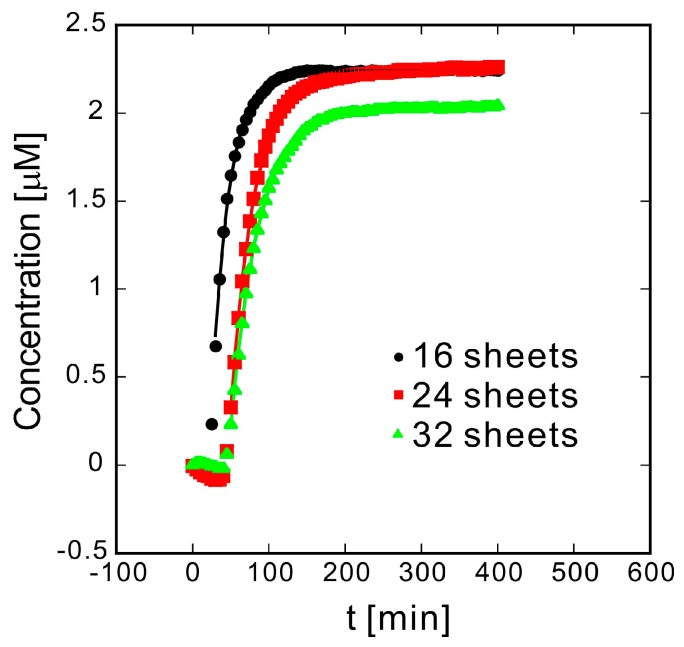
Time dependence of methylene blue absorbance at 664 nm for different quantities of laminated sheets. The markers indicate experimental results, and the solid line is the result of fitting the experimental data to the exponential function (*m*_1_ × (1 − e^(−*m*2 × (*t*−*m*3))^) using the least squares method.

The correlation between elution distance and elution rate was investigated using samples prepared by changing the number of sheets and sample thickness simultaneously to obtain similar porosities. The absorbance is converted to solution concentration, *C*_bulk_, which is fitted to an exponential increase equation (Figure 4). The range of the best-fit line (solid line) is the range of the fitted data, which matches the experimental results well. The fitting equation is as follows:

(1) $$C_{\mathrm{bulk}}=m_{1}\left(1-\exp\left(-m_{2}\times \left(t-m_{3}\right)\right)\right)$$

where *m*_1_ is the equilibrium concentration, *m*_2_ is the apparent elution rate, *m*_3_ is the apparent delay time, and *t* is the time. The delay time is the time taken by the buffer solution to penetrate the sample and reach the methylene-blue-loaded fabric and for methylene blue to diffuse from the sample.

Because methylene blue did not remain in the sample after the elution test, it was assumed that all of it diffused into the bulk. This suggests that there is no special interaction between methylene blue and the nonwoven fabric.

*C*_bulk_ rises exponentially at rate *m*_2_ after a certain time *m*_3_ and eventually approaches *m*_1_. As the number of sheets *n* increases and the elution distance lengthens, *m*_2_ becomes slower (Table 1). Although the samples were prepared similarly, there is a slight variation in *m*_1_. This is because while methylene blue is placed on the nonwoven fabric, part of it remains on the PTFE sheet (Figure 1) depending on the sample, leading to a difference in the amount of methylene blue loaded. 

In this experiment, samples are slowly circulated between the beaker and optical cell by the peristaltic pump (8.8 mL h^−1^), and a time difference occurs between the change in concentration in the beaker and reflection for the optical cell. Therefore, the time constant of the experimental set-up was estimated. After methylene blue was dropped in the beaker, the time dependence of the absorbance was measured with the dropping time set to 0, and the response of the optical cell due to the instantaneous rise of the concentration in the beaker, as well as an exponential rise in delay time, was observed. The delay time at this time was *m*_3_′ = 6.73 min and elution rate *m*_2_′ was 0.1623 min^−1^. When both the behavior of elution and observed reflection are exponential, there is a difference between the true elution rate, *m*_2_″, and *m*_2_, and true delay time, *m*_3_″, and *m*_3_. The differences are described as follows:

(2) $$m_{2}=\frac{m_{2}^{\prime }m_{2}^{\prime \prime }}{m_{2}^{\prime }+m_{2}^{\prime \prime }}$$

(3) $$m_{3}=m_{3}^{\prime }+m_{3}^{\prime \prime }$$

When the observed delay time is short (*m*_3_′ << *m*_3_″) and the reflection rate is high (*m*_2_′ >> *m*_2_″), *m*_3_″ and *m*_3_, and *m*_2_″ and *m*_2_ are in agreement. However, in this experiment, these conditions were not satisfied. Thus, it was necessary to calculate *m*_2_″ and *m*_3_″ from Equations (4) and (5).

(4) $$m_{2}^{\prime \prime }=\frac{m_{2}m_{2}^{\prime }}{m_{2}^{\prime }-m_{2}}$$

(5) $$m_{3}^{\prime \prime }=m_{3}-m_{3}^{\prime }$$

**Figure 5:**
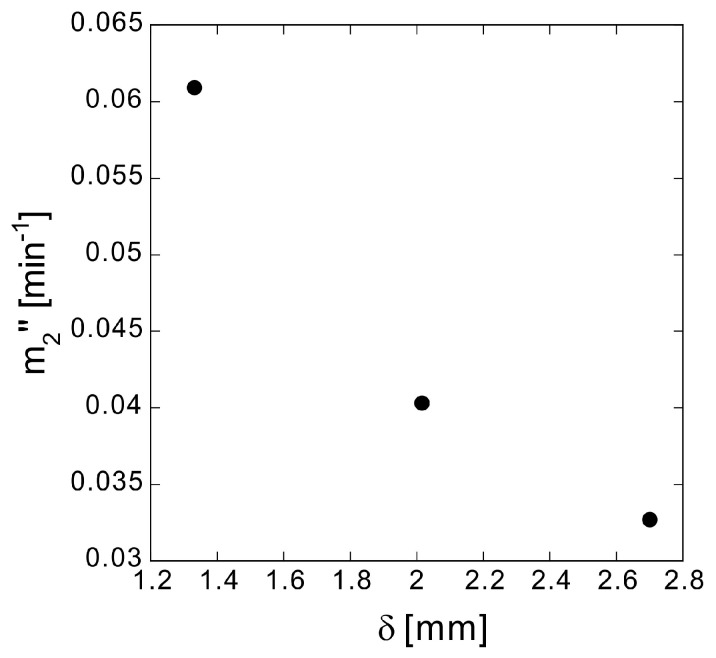
True elution rate constant *m*_2_″ at different sample thicknesses *δ*.

The plot of the derived values in Figure 5 show that *m*_2_″ decreases as *δ* increases.

**Figure 6:**
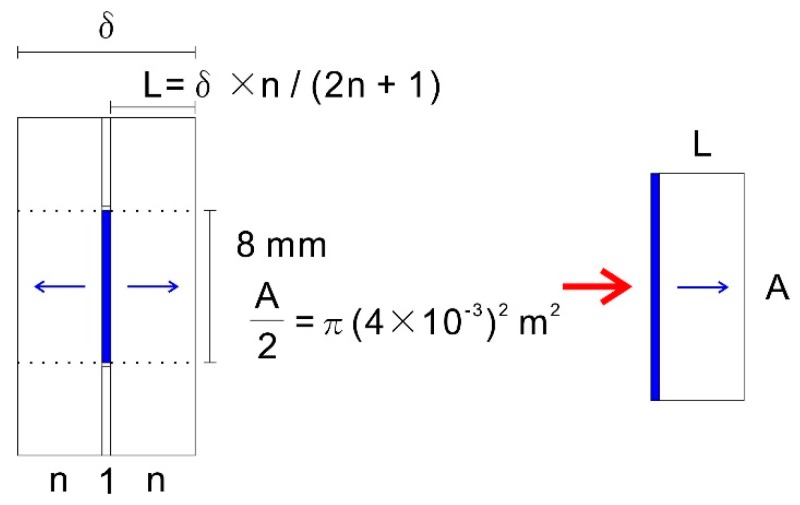
Diffusion model for the release of the drug from the tablet.

The effective diffusion coefficient in the porous material (*D*_eff_) of the tablet was investigated by constructing a diffusion model. In this model, the state after delay time *m*_3_″ associated with the initial penetration of water, as seen in the experiment, was set to time 0. In addition, to simplify the model, unidirectional diffusion behavior in a porous material with an area twice the elution cross-section was assumed instead of gradual release of the drug at the center from both sides of the tablet (Figure 6). The thickness of the diffusion layer (*L*) was calculated from the number of sheets (*n*) on each side, total number of sheets (2*n* + 1), and sample thickness *δ*, assuming that the overlapping nonwoven fabrics are evenly compressed (Figure 6).

The driving force for the diffusion of solute in solution is the concentration difference, and in describing the diffusion behavior in the tablet, estimation of the concentration inside the tablet (*C*_drug_) is indispensable. Experimental results showed that the concentration outside the tablet is *C*_bulk_, which is 0 at time 0 and *m*_1_ at time ∞. As shown above, *C*_bulk_ rose exponentially. On the basis of this information, we attempted to formulate a model. First, it was assumed that *C*_drug_ at time 0, which is unknown, is *C*_0_. At time ∞, *C*_drug_ coincides with *C*_bulk_, and the release of the drug, with concentration as the driving force, stops. When the amount of solute introduced into the porous material and the amount of solute discharged from the porous material are equivalent, the decay of *C*_drug_ should be exponential, since the change in *C*_bulk_ after passing through the porous material is also exponential. Thus, it is necessary to link *C*_drug_(0) = *C*_0_ and *C*_drug_(∞) = *m*_1_ exponentially. Then, under the condition that the amount of solute introduced into the porous material is equal to the amount of solute discharged from the porous material, the rate constant for the decrease in *C*_drug_ must match the rate constant for the increase in *C*_bulk_. Based on this hypothesis, the following equation was derived:

(6) $$C_{drug}=m_{1}+\left(C_{0}-m_{1}\right)\exp\left(-m_{2}^{\prime \prime }t\right)$$

Table 2 summarizes the concentration difference between the interior of the tablet and the bulk, which is the driving force for diffusion.

The change in diffusion flow rate *J* was calculated from the amount of solute eluted from the tablet. If the bulk amount of water is *V*_bulk_ and the pores in the tablet are *V*_void_, the bulk amount of water after the water has seeped into the pores is (*V*_bulk_ – *V*_void_). Sample c in Table 1 has the largest *V*_void_ among the samples; *V*_void_ is about 0.3 mL (0.9 × 0.9 × 3.14 × 0.2701 × 0.453), which is negligible compared to *V*_bulk_ in the beaker (200 mL). That is, the space inside the tablet is very small relative to *V*_bulk_ (*V*_bulk_ >> *V*_void_); thus, in this experiment, *V*_bulk_ can be considered constant at 200 mL. Under this condition, by multiplying the *C*_bulk_ (mol m^−3^) by *V*_bulk_ (200 mL = 2 × 10^−4^ m^3^), the amount of methylene blue eluted from the tablet into bulk water can be obtained. Under the condition that the concentration gradient in the porous material can be approximated linearly, *J* is given by

(7) $$J=\frac{D_{eff}\left(C_{drug}-C_{bulk}\right)}{L}=\frac{D_{eff}C_{0}exp\left(-m_{2}^{\prime \prime }\right)}{L}$$

Thus, *D*_eff_ can be calculated using the equation

(8) $$D_{eff}=\frac{L\;J}{C_{0}exp\left(-m_{2}^{\prime \prime }t\right)}$$

On the other hand, since the elution rate is obtained by differentiating the amount eluted into bulk water (2 × 10^−4^ *C*_bulk_) by *t*, J is given by

(9) $$J=\frac{2\times 10^{-4}m_{1}m_{2}^{\prime \prime }\exp\left(-m_{2}^{\prime \prime }t\right)}{A}$$

From Equations (8) and (9), *D*_eff_ is derived as follows:

(10) $$D_{eff}=\frac{2\times 10^{-4}m_{1}m_{2}^{\prime \prime }L}{AC_{0}}$$

**Figure 7:**
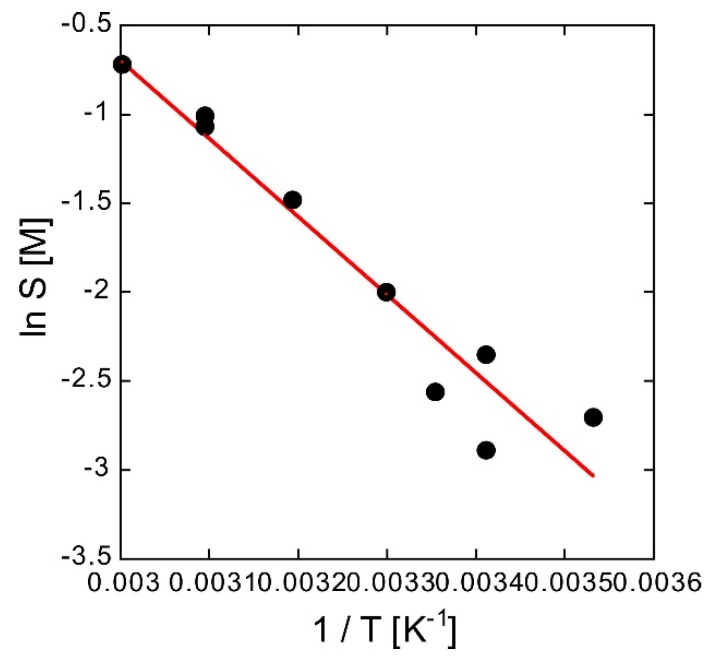
Van’t Hoff plot of solubility *S* of methylene blue in 1 mM MES buffer solution.

Normally, since the unit of *D*_eff_ is m^2^ s^−1^, it is necessary to convert the units of *m*_1_ to mol m^−3^, *m*_2_″ to s^−1^, *L* to m, *A* to m^2^, and *C*_0_ to mol m^−3^. Usually, elution from solid drugs is indicated by elution of the surface of the drug into the solvent and diffusion after elution. For the concentration near the surface of the drug, the saturation solubility should be used as *C*_0_. The solubility *S* was determined using a van’t Hoff plot with 1/*T* on the abscissa and ln *S* on the ordinate. In Figure 7, the markers are the measured values and the solid line is the linear fitting obtained by the least squares method. To suppress fluctuations in the experiments, subsequent analysis was performed using the *S* obtained from the fitting of the van’t Hoff plot. Data fitting using the least squares method yields the following equation:

(11) $$\ln\;S=12.49-\frac{4394}{T}$$

The *S* in the aqueous solution containing 1 mM MES buffer at 25 °C, calculated from Equation (11), was 0.105 M. This is close to the solubility in water at 25 °C stated in the SDS sheet of the methylene blue reagent, which is 0.136 M. 

The results of the calculation of *D*_eff_ in the porous body are shown in Table 3. It is known that *D*_eff_ in a porous body filled with solvent is smaller than the bulk diffusion coefficient. This is because the diffusion path is narrower if the solvent exists only in the gap of the porous material (effect of porosity). Furthermore, the path in the gap of the porous body is bent and the diffusion path is longer than *L* (effect of tortuosity). Assuming that the bulk diffusion coefficient is *D*_bulk_, *D*_eff_ is given by the following equation [22].

(12) $$D_{eff}=\frac{\alpha }{\tau }D_{bulk}$$

where *τ* is the tortuosity factor. For one-dimensional diffusion, *τ* is defined as the square of *L*_eff_/*L*, where *L*_eff_ is the actual diffusion distance. The diffusion coefficient of methylene blue in water at 23 °C is 4.6 × 10^−10^ m^2^ s^−1^ according to Miložič et al. [23]. The arbitrary temperature can be estimated in proportion to the absolute temperature using the Einstein-Stokes equation. *D*_bulk_ at 25 °C is 4.6 × 10^−10^ m^2^ s^−1^, and the calculated *τ* values using this and Equation (12) are shown in Table 3. The *D*_eff_ and *τ* values of samples a, b, and c are same; thus, the decrease in *m*_2_″ with increasing *δ* is caused by the expansion of the diffusion distance.

**Figure 8:**
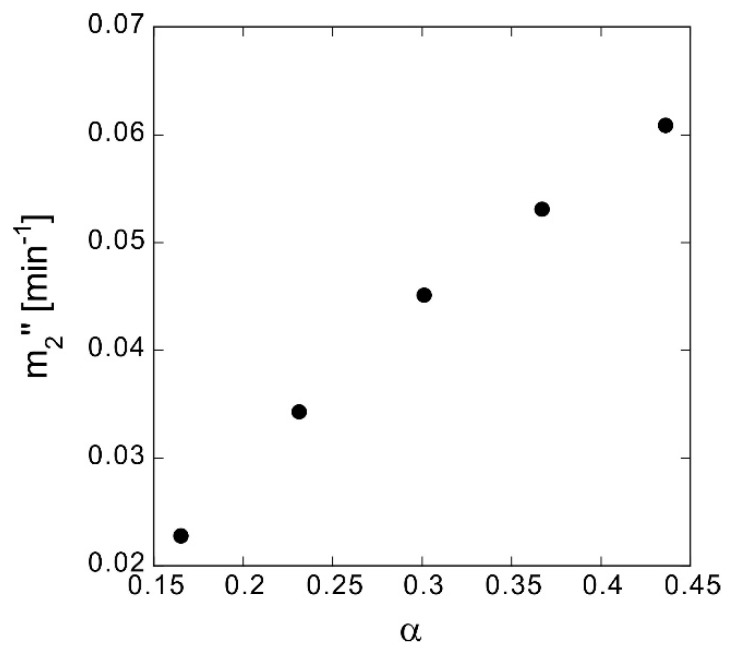
True elution rate *m*_2_″ at different porosities *α*.

Subsequently, without changing *δ*, samples with different compression ratios and associated *α* were produced by increasing the number of overlapping sheets. From samples a to g in Table 4, the number of sheets on one side (*n*) increases by two and *α* decreases. *m*_2_″ increases as *α* increases (Figure 8) and since *δ* is almost the same, *D*_eff_ also decreases (Table 4). The calculated *τ* values of samples a, d, e, and f are almost the same, but that of sample g is higher; this may be due to the influence of sample fluctuation. It is natural that *α* decreases as the compression ratio increases and that *τ* shows a tendency to increase. Nevertheless, the difference in *D*_eff_ is considerably larger than the difference in *τ*, and the former is dominated by the decrease in the volume involved in elution caused by the decrease in *α*.

**Figure 9:**
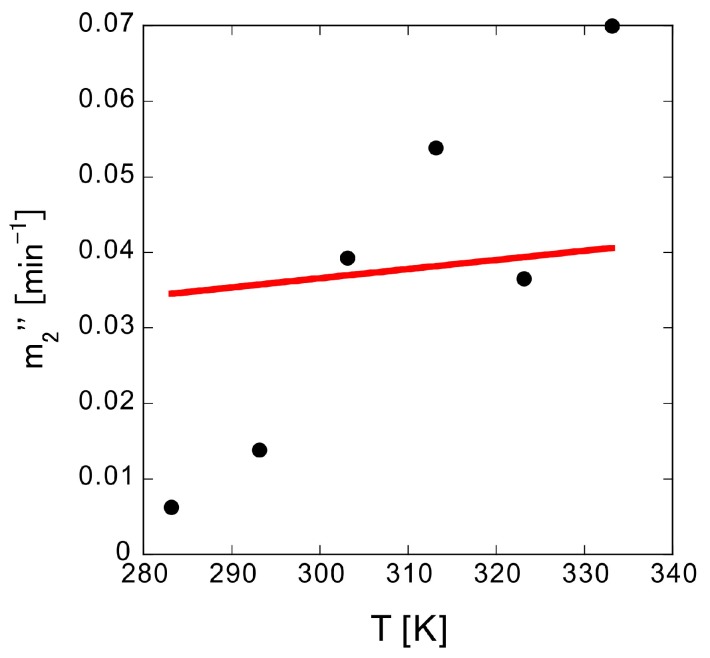
Temperature dependence of true elution rate *m*_2_″. The circles are experimental data, while the red solid line is the proportional fitting.

Finally, the temperature dependence of *m*_2_″ was measured. The *m*_2_″ values of samples h to m, prepared with 20 sheets pressed on one side, were measured from 10 to 60 °C. If the diffusion coefficient follows the Einstein-Stokes equation, it is proportional to the absolute temperature; however, the temperature dependence of *m*_2_″ is not proportional to the absolute temperature (Figure 9). Thus, factors other than the diffusion coefficient should dominate the trend observed for *m*_2_″. Analysis based on the tablet elution model was consequently carried out.

**Figure 10:**
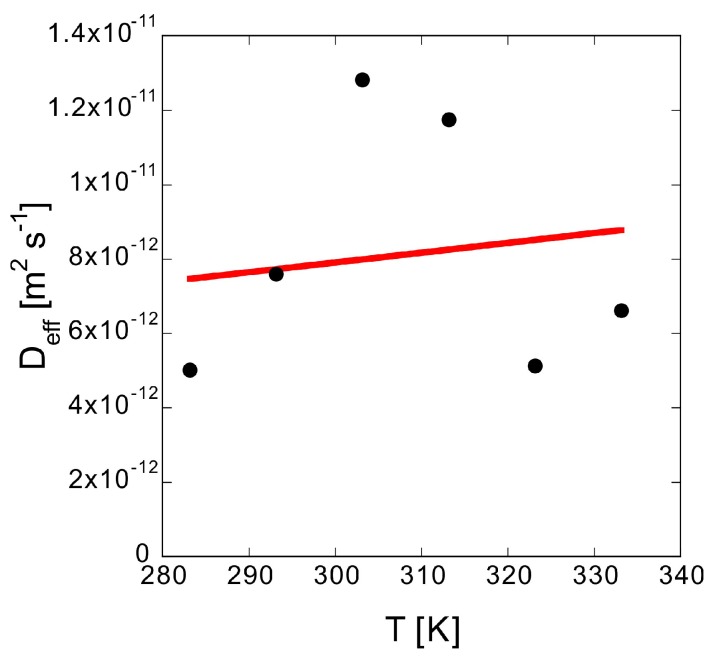
Temperature dependence of *D*_eff_. The circles are experimental data, while the red solid line is the proportional fitting.

Figure 10 shows that *D*_eff_ gradually increases with *T*. According to the Einstein-Stokes equation, *D*_bulk_ is proportional to *T*. For a porous material with the same *α* and degree of flexion, *D*_eff_ should also be proportional to *T*; thus, the solid line from the least squares approximation shows proportionality. Although the data fluctuation is large, the trends generally agree. *m*_2_″ varies significantly with *T* despite the moderate change in *D*_eff_. This is because the *S* of methylene blue varies significantly with *T* (Table 5). With the concentration difference as the driving force, *J* increases, leading to an increase in *m*_2_″.

## 4. Conclusions

In this study, various drug-containing tablets were prepared and their controlled release was analyzed by determining the effects of thickness, porosity, and temperature. A diffusion model that assumes an exponential decrease in the drug concentration inside the tablet from the saturated solubility was formulated. The drug placed inside the nonwoven fabric would diffuse through various paths from the interior to the surface. When medicine carried on a nonwoven fabric is used as a drug supply, it is assumed that the drug on the surface would be released earlier, while that inside would be gradually released. The drug inside goes through the nonwoven fabric and reaches the surface, and this can be considered an exponential decrease in the drug supply. 

The proposed diffusion model of the drug-loaded tablet explained the experimental data well. The experiments showed that thickness and porosity significantly affected sustained release performance. Using the model to calculate the effective diffusion coefficient and tortuosity factor showed that a decrease in porosity did not significantly increase the tortuosity factor. In the experiments, the decrease in the diffusion volume was the dominant factor in the change in the effective diffusion coefficient of the porous material.

We verified that the model can predict the sustained release rate through fitting experiment data for the elution behavior. The effective diffusion coefficient can be calculated from the sustained release rate; conversely, when the effective diffusion coefficient of a sample is known, the sustained release rate can be calculated using the model. In other words, it is possible to use the proposed diffusion model to design tablets that can realize a specific sustained release rate.

## Figures and Tables

**Table 1:** Thickness *δ* and porosity *α* of samples with *n* sheets and related parameters ^1^ of methylene blue elution.

ID	*n*	Weight of *n* Sheets (g)	*δ* (mm)	*α*	*m*_1_ (μM)	*m*_2_ (min^−1^)	*m*_3_ (min)
a	16	0.124	1.331	0.436	2.24	0.0443	21.0
b	24	0.186	2.015	0.447	2.25	0.0323	45.3
c	32	0.248	2.701	0.453	2.09	0.0272	45.9

^1^*m*_1_, equilibrium concentration; *m*_2_, apparent elution rate; *m*_3_, apparent delay time.

**Table 2:** Concentrations inside (*C*_drug_) and outside (*C*_bulk_) the tablet at different times.

Time	*C*_drug_^1^	*C*_bulk_
0	*C*_0_	0
t	*m*_1_ + (*C*_0_ − *m*_1_)exp(−*m*_2″_*t*)	*m*_1_(1 − exp(−*m*_2″_*t*))
∞	*m*_1_	*m*_1_

^1^*C*_0_, concentration at t = 0; *m*_1_, equilibrium concentration; *m*_2″_, true elution rate constant.

**Table 3:** Effective diffusion coefficient *D*_eff_ and tortuosity factor *τ* of samples and related parameters ^1^ of methylene blue elution.

ID	*δ* (mm)	*α*	*m*_2_″ (min^−1^)	*m*_3_″ (min)	*D*_eff_ (m^2^ s^−1^)	*τ*	*L*_eff_/*L*
a	1.331	0.436	0.0609	14.3	2.8 × 10^−11^	7.3	2.7
b	2.015	0.447	0.0403	38.6	2.8 × 10^−11^	7.3	2.7
c	2.701	0.453	0.0327	39.2	2.9 × 10^−11^	7.3	2.7

^1^*δ*, sample thickness; *α*, porosity; *m*_2″_, true elution rate constant; *m*_3″_, true delay time; *L*, thickness of diffusion layer; *L*_eff_, actual diffusion distance.

**Table 4:** Effect of compression ratio on parameters ^1^ of methylene blue elution.

ID	*n*	Weight of *n* Sheets (g)	*δ* (mm)	*α*	*m*_2_″ (min^−1^)	*D*_eff_ (m^2^ s^−1^)	*τ*	*L*_eff_/*L*
a	16	0.124	1.331	0.436	0.0609	2.8 × 10^−11^	7.3	2.7
d	18	0.140	1.333	0.367	0.0531	2.3 × 10^−11^	7.3	2.7
e	20	0.155	1.333	0.301	0.0451	2.0 × 10^−11^	7.1	2.7
f	22	0.171	1.335	0.231	0.0343	1.6 × 10^−11^	6.7	2.6
g	24	0.180	1.335	0.165	0.0228	8.4 × 10^−12^	9.1	3.0

^1^*δ*, sample thickness; *α*, porosity; *m*_2″_, true elution rate; *D*_eff_, effective diffusion coefficient; *τ*, tortuosity factor; *L*, thickness of diffusion layer; *L*_eff_, actual diffusion distance.

**Table 5:** Effect of temperature on parameters ^1^ of methylene blue elution.

ID	*T* (°C)	Weight of *n* Sheets (g)	*δ* (mm)	*α*	*m*_2_″ (min^−1^)	*S* (M)	*D*_bulk_ (m^2^ s^−1^)	*D*_eff_ (m^2^ s^−1^)	*τ*	*L*_eff_/*L*
h	10	0.155	1.130	0.170	0.00604	0.048	4.4 × 10^−10^	5.0 × 10^−12^	15	3.9
i	20	0.155	1.132	0.171	0.0127	0.082	4.6 × 10^−10^	7.6 × 10^−12^	10	3.2
j	30	0.155	1.133	0.172	0.0316	0.13	4.7 × 10^−10^	1.3 × 10^−11^	6.3	2.5
k	40	0.155	1.128	0.168	0.0404	0.21	4.9 × 10^−10^	1.2 × 10^−11^	7.0	2.6
l	50	0.155	1.129	0.169	0.0298	0.33	5.0 × 10^−10^	5.1 × 10^−12^	17	4.1
m	60	0.155	1.131	0.170	0.0489	0.94	5.2 × 10^−10^	6.6 × 10^−12^	13	3.6

^1^*δ*, sample thickness; *α*, porosity; *m*_2″_, true elution rate constant; *S*, solubility; *D*_bulk_, diffusion coefficient in bulk water; *D*_eff_, effective diffusion coefficient; *τ*, tortuosity factor; *L*, thickness of diffusion layer; *L*_eff_, actual diffusion distance.
